# Predicting early reconnection after cryoballoon ablation with procedural and biophysical parameters

**DOI:** 10.1016/j.hroo.2021.03.007

**Published:** 2021-03-19

**Authors:** Fehmi Keçe, Marta de Riva, Reza Alizadeh Dehnavi, Adrianus P. Wijnmaalen, Bart J. Mertens, Martin J. Schalij, Katja Zeppenfeld, Serge A. Trines

**Affiliations:** ∗ Department of Cardiology, Heart Lung Center, Leiden University Medical Center, Leiden, The Netherlands; † Bioinformatics Center of Expertise, Leiden University Medical Center, Leiden, The Netherlands

**Keywords:** Atrial fibrillation, Cryoballoon ablation, Dormant conduction, Pulmonary vein isolation, Time-to-isolation

## Abstract

**Background:**

Predicting early reconnection/dormant conduction (ERC) immediately after pulmonary vein isolation (PVI) can avoid a waiting period with adenosine testing.

**Objective:**

To identify procedural and biophysical parameters predicting ERC.

**Methods:**

Consecutive atrial fibrillation (AF) patients undergoing a first cryoballoon ablation (Arctic Front Advance) between 2014 and 2017 were included. ERC was defined as manifest or dormant pulmonary vein (PV) reconnection with adenosine 30 minutes after PVI. Time to isolation (TTI), balloon temperatures (BT), and thawing times were evaluated as potential predictors for ERC. Based on a multivariable model, cut-off-values were defined and a formula was constructed to be used in clinical practice.

**Results:**

A total of 136 patients (60 ± 10 years, 96 male, 95% paroxysmal AF) were included. ERC was found in 40 (29%) patients (ERC group) and in 53 of 575 (9%) veins. Procedural and total ablation time and the number of unsuccessful freezes were significantly longer/higher in the ERC group compared to the non-ERC group (150 ± 40 vs 125 ± 34 minutes; 24 ± 5 vs 17 ± 4 minutes, and 38% vs 24%, respectively (*P* = .028). Multivariable analysis showed that a higher nadir balloon temperature (hazard ratio [HR] 1.17 [1.09–1.23, *P* < .001), a higher number of unsuccessful freezes (HR 1.69 [1.15–2.49], *P* = .008) and a longer TTI (HR 1.02 [1.01–1.03], *P* < .001) were independently associated with ERC, leading to the following formula: 0.02 × TTI + 0.5 × number of unsuccessful freezes + 0.2 × nadir BT with a cut-off value of ≤-6.7 to refrain from a waiting period with adenosine testing.

**Conclusion:**

Three easily available parameters were associated with ERC. Using these parameters during ablation can help to avoid a 30-minute waiting period and adenosine testing.

**Key Findings**

▪ Early reconnection/dormant conduction can be seen in 29% of the patients and 9% of the pulmonary veins after cryoballoon pulmonary vein isolation.
▪ Biophysical parameters associated with early reconnection and dormant conduction are a higher nadir balloon temperature, a higher number of unsuccessful freeze attempts, and a longer time to isolation.
▪ Based on the cut-off values of the 3 parameters, we constructed a prediction model for early reconnection/dormant conduction with a negative predictive value of 97%. Applying our model, operators can decide to refrain from the 30-minute waiting protocol and adenosine testing in certain patients.

## Introduction

Cryoballoon ablation is an effective treatment for drug-resistant atrial fibrillation (AF) owing to its ease of use, its low complication rates, and shorter procedural times compared to radiofrequency catheter ablation.^1^ AF recurrence rates after cryoballoon ablation can be decreased if a 30-minute waiting period for detecting early pulmonary vein (PV) reconnection is applied, followed by testing with adenosine for identifying dormant conduction.^2^, ^3^, ^4^, ^5^, ^6^ However, this approach is time-consuming and is contraindicated in patients with reactive airway disease or atrioventricular conduction disorders. Therefore, predicting the absence of early PV reconnection and dormant conduction immediately after a cryoballoon application would be desirable.

In a prior study, a time to isolation ≤60 seconds and a thawing time to 0°C of ≥10 seconds during the index cryoablation were associated with durable PV isolation assessed during a repeat ablation 14 ± 3 months after the index procedure.^7^ However, sites of early (after 30-minute) reconnection may differ from sites of late (repeated-procedure) reconnection^6^ and biophysical data associated with late reconnection may not predict early reconnection/dormant conduction (ERC).

The purpose of this study was therefore to identify procedural and biophysical parameters to predict the absence of ERC at the time of the index procedure.

## Methods

### Study population

Consecutive patients undergoing a first AF ablation with cryoballoon at the Leiden University Medical Center between 2014 and 2017 were included. Patient characteristics and procedural data were collected using the departmental Cardiology Information System (EPD-Vision). The ablation files were extracted from the ablation console to derive biophysical parameters of each cryo-application. This study was approved by the institutional ethical review board. A written informed consent was not necessary for this retrospective study. The research in this study was conducted in accordance with the guidelines outlined in the Declaration of Helsinki.

### Ablation procedure

All antiarrhythmic drugs (except amiodarone) were discontinued for at least 3 days before ablation. Ablation was performed with a 28 mm second-generation cryoballoon (Arctic Front Advance; Medtronic Inc, Minneapolis, MN). The 23 mm balloon was only used in PVs with a diameter <20 mm in which PV isolation could not be achieved with the 28 mm balloon. A single cryo-application was performed per PV. The ablation duration was set to 240 seconds except for the right superior PV, in which the application duration was decreased to 180 seconds to prevent phrenic nerve palsy.^2^^,^^8^ During ablation, time to isolation was measured, defined as the time from start of the application until disappearance of the PV potentials recorded from a 20 mm intraluminal circumferential mapping catheter with 8 electrodes (Achieve; Medtronic, Minneapolis, MN). The cryo-application was aborted if isolation was not achieved within 90 seconds. Subsequently, the balloon was repositioned in an attempt to improve PV occlusion and to achieve PV isolation within 90 seconds. After a waiting period of 30 minutes, PV isolation was reassessed. If a given PV was reconnected, additional cryo-applications were performed until PV isolation (PVI) was achieved. In the presence of PVI, dormant PV conduction was tested during adenosine infusion. An increasing dose of adenosine (18 up to 30 mg intravenous) was administered until >1 sinus beat with blocked AV conduction was observed. In case of dormant conduction, additional applications were performed, with a maximum of 2. Early reconnection was defined as acute reconnection directly after the application or reconnection or dormant conduction tested with adenosine after a waiting period of 30 minutes. For the prevention of phrenic nerve palsy, high-output pacing (20 mA/2 ms) of the phrenic nerve from the superior caval vein was performed with manual palpation of the diaphragmatic movement to confirm and control capture. Endoluminal esophageal temperature was monitored with a nasal temperature probe (Sensitherm; St. Jude Medical, Saint Paul, MN). Applications were terminated with a “double stop technique” if the temperature of the esophagus reached <18°C or a reduced diaphragmatic movement was observed. Procedural characteristics including number of applications and unsuccessful freezes per vein (defined as aborted applications because of absence of PV isolation within 90 seconds) and time to isolation as well as biophysical data were evaluated as potential predictors for ERC.^7^

### Follow-up

Patients were followed 3, 6, and 12 months after ablation with a 24-hour Holter monitor and an exercise test. In addition, all symptomatic patients were encouraged to immediately visit the outpatient clinic for rhythm documentation on 12-lead ECGs or additional 24-hour Holter registration during palpitations. After the procedure, antiarrhythmic drugs were restarted and stopped at the first follow-up visit if no AF/atrial tachycardia recurrence was documented. After a blanking period of 3 months, ablation success was defined as the absence of any documentation of AF/atrial tachycardia lasting longer than >30 seconds on ECG, Holter, or device recording.

### Statistical analysis

Categorical variables were compared using the χ^2^ test (or Fisher exact test when appropriate) and continuous variables with the independent *t* test (or Mann-Whitney *U* test). Predictors of ERC were identified by multivariable logistic regression using variables with statistically significant differences in the univariable analysis between the groups. This resulted in a model providing odds ratios and 95% confidence intervals for the primary outcome. A receiver operating characteristics (ROC) curve corresponding to the selected logistic regression was constructed and the area under the curve was calculated to provide a summary measure of the accuracy of the prediction model. Based on the individual coefficients of the regression model a formula was created and based on the ROC curve, a combined cut-off value was determined for the significant parameters. Finally, the AF-free survival was compared between groups using the log-rank test. A *P* value of <.05 was considered statistically significant. R-studio (Version 1.0.143 – © 2009-2016 RStudio, Inc) was used for data extraction and calculation of biophysical data from the console files and SPSS (version 23; SPSS Inc, Chicago, IL) was used for data analysis.

## Results

### Baseline characteristics

A total of 151 patients (60 ± 9 years, 108 male, 95% paroxysmal AF) were included. Fifteen patients and 29 veins were excluded from the analysis, because dormant conduction testing was not performed owing to contraindications for adenosine (asthma). The final population consisted of 136 patients, and 575 veins were analyzed. ERC was found in 40 (29%) patients (ERC group) and in 53 (9%) veins. ERC was more prevalent in male patients (83% vs 66%, *P* = .049). Other baseline clinical characteristics were comparable between the groups (Table 1).

**Table 1:** Baseline characteristics

	Overall (n = 136)	No ERC (n = 96)	ERC (n = 40)	*P* value
Age (y), mean	60 ± 10	60 ± 10	60 ± 9	.789
Male sex, n (%)	96 (71%)	63 (66%)	33 (83%)	.049
AF duration, years	4.3 ± 3.5	4.4 ± 3.8	3.9 ± 3.1	.473
CHA_2_DS_2_-VASc score	1.5 ± 1.3	1.6 ± 1.4	1.4 ± 1.2	.333
Body mass index, kg/m^2^	27.0 ± 3.8	26.9 ± 3.8	27.3 ± 3.8	.538
AAD at baseline, n (%)	115 (85%)	82 (85%)	33 (83%)	.668
Amiodarone	19 (14%)	14 (15%)	5 (13%)	.749
Paroxysmal AF	129 (95%)	93 (97%)	36 (90%)	.098
**Comorbidity**				
Hypertension, n (%)	57 (42%)	44 (46%)	13 (33%)	.151
Dyslipidemia, n (%)	39 (29%)	24 (25%)	15 (38%)	.142
Diabetes, n (%)	11 (8%)	6 (6%)	5 (135%)	.300
Sleep apnea, n (%)	9 (7%)	5 (5%)	4 (10%)	.449
Coronary artery disease, n (%)	19 (14%)	13 (14%)	6 (15%)	.823
Structural heart disease, n (%)	9 (7%)	6 (6%)	3 (8%)	.722
**Imaging**				
LA diameter (mm), mean, SD	41 ± 4	40 ± 4	41 ± 5	.706

AAD = antiarrhythmic drugs; AF = atrial fibrillation; LA: left atrium.

### Procedural details

Procedure and total ablation time were longer in the ERC group compared to the non-ERC group (150 ± 40 minutes vs 125 ± 34 minutes and 24 ± 5 minutes vs 17 ± 4 minutes; both *P* < .001). The total number of applications (8 ± 2 vs 5 ± 1, *P* < .001) and the number of unsuccessful freezes (38% vs 24%, *P* = .028) of the PVs were significantly higher in the ERC group. Time to isolation could be measured in 80% of the PVs during ablation and was significantly longer in the ERC group (70 ± 30 seconds vs 48 ± 28 seconds). Procedural details are displayed in Table 2.

**Table 2:** Procedural characteristics

Per patient or per vein	Overall (n = 136/575)	No ERC (n = 96/522)	ERC (n=40/53)	*P* value
Procedure time (min), mean	133 ± 37	125+34	150+40	<.001
Total ablation time (min), mean	19 ± 5	17 ± 4	24 ± 5	<.001
Number of applications per patient, mean	6 ± 2	5 ± 1	8 ± 2	<.001
Number of applications per vein, mean	1.4 ± 0.8	1.3 ± 0.7	1.7 ± 1.2	.012
LSPV	1.4 ± 0.8	1.3 ± 0.8	2.1 ± 1.5	.048
LIPV	1.5 ± 0.8	1.4 ± 0.7	1.9 ± 1.3	.120
RSPV	1.2 ± 0.4	1.1 ± 0.4	1.3 ± 0.5	.135
RIPV	1.5 ± 1.0	1.5 ± 1.0	1.5 ± 0.9	.968
Absence of PVI within 90 seconds of index ablation n (%)	114 (20%)	104 (20%)	10 (19%)	.854
LSPV (n = 145)	22 (15%)	20 (15%)	2 (17%)	.573
LIPV (n = 140)	17 (12%)	16 (13%)	1 (6%)	.694
RSPV (n = 145)	30 (21%)	27 (20%)	3 (39%)	.342
RIPV (n = 145)	45 (31%)	41 (31%)	4 (29%)	.550
Balloon size (28 mm), n (%)	127 (93%)	89 (93%)	37 (83%)	1.000
Balloon size (23 mm), n (%)	5 (4%)	5 (5%)	1 (3%)	.670
Balloon size (23 and 28 mm), n (%)	4 (3%)	2 (2%)	2 (5%)	.581
Effective radiation dose (mSV), mean	2.9 ± 2.6	2.7 ± 2.6	3.5 ± 2.5	.081
Time to isolation (s)	50 ± 29	48 ± 28	70 ± 39	.001
Total ablation time (s)	214 ± 41	213 ± 41	219 ± 43	.302
Minimal esophagus temperature (°C)	33 ± 6	33 ± 6	31 ± 12	.254
Unsuccessful freezes, n (%)	145 (25%)	125 (24%)	20 (38%)	.028
Aborted freezes, n (%)	64 (11%)	56 (11%)	8 (15%)	.336

LIPV = left inferior pulmonary vein; LSPV = left superior pulmonary vein; PVI = pulmonary vein isolation; RIPV = right inferior pulmonary vein; RSPV = right superior pulmonary vein.

### Biophysical data

The balloon temperature at 60 seconds (-35°C ± 6°C vs -39°C ± 6°C, *P* = .004) and the nadir balloon temperature were significantly lower in the non-ERC-group (-42°C ± 9°C vs -47°C ± 7°C, *P* < .001). In addition, significantly shorter thawing times were achieved at 0°C, 15°C, and 20°C in the ERC group. The mean balloon temperature below 0°C was -35°C ± 7°C in the ERC group compared to -38°C ± 5°C in the non-ERC group (*P* < .001). In Table 3, an overview of all biophysical parameters is shown.

**Table 3:** Biophysical parameters

Per vein	Overall (n = 575)	No ERC (n = 522)	ERC (n = 53)	*P* value
Temperature at 30 seconds (°C)	-31 ± 6	-31 ± 5	-29 ± 8	.058
Temperature at 60 seconds (°C)	-38 ± 6	-39 ± 6	-35 ± 8	.004
Nadir balloon temperature (°C)	-47 ± 7	-47 ± 7	-42 ± 9	<.001
Temperature at time to isolation (°C)	-34 ± 8	-34 ± 8	-34 ± 8	.789
Freeze (AUC)	7928 ± 1797	7999 ± 7285	7285 ± 1961	.007
Freeze magnitude (freeze AUC/total application time)	38 ± 5	38 ± 5	35 ± 7	<.001
Warming time to 0°C (s)	9 ± 6	9 ± 6	6 ± 4	.001
Warming time to 15°C (s)	34 ± 16	35 ± 16	25 ± 16	<.001
Warming time to 20°C (s)	41 ± 18	42 ± 18	31 ± 18	<.001

AUC = area under the curve (sum of temperature × time).

### Early reconnection/dormant conduction

Reconnection without adenosine was seen in 28 (19%) of the patients and 30 (5%) of the veins, while dormant conduction was observed in 21 (14%) patients and 28 (4%) veins.

### Predictors of ERC

**Figure 1:**
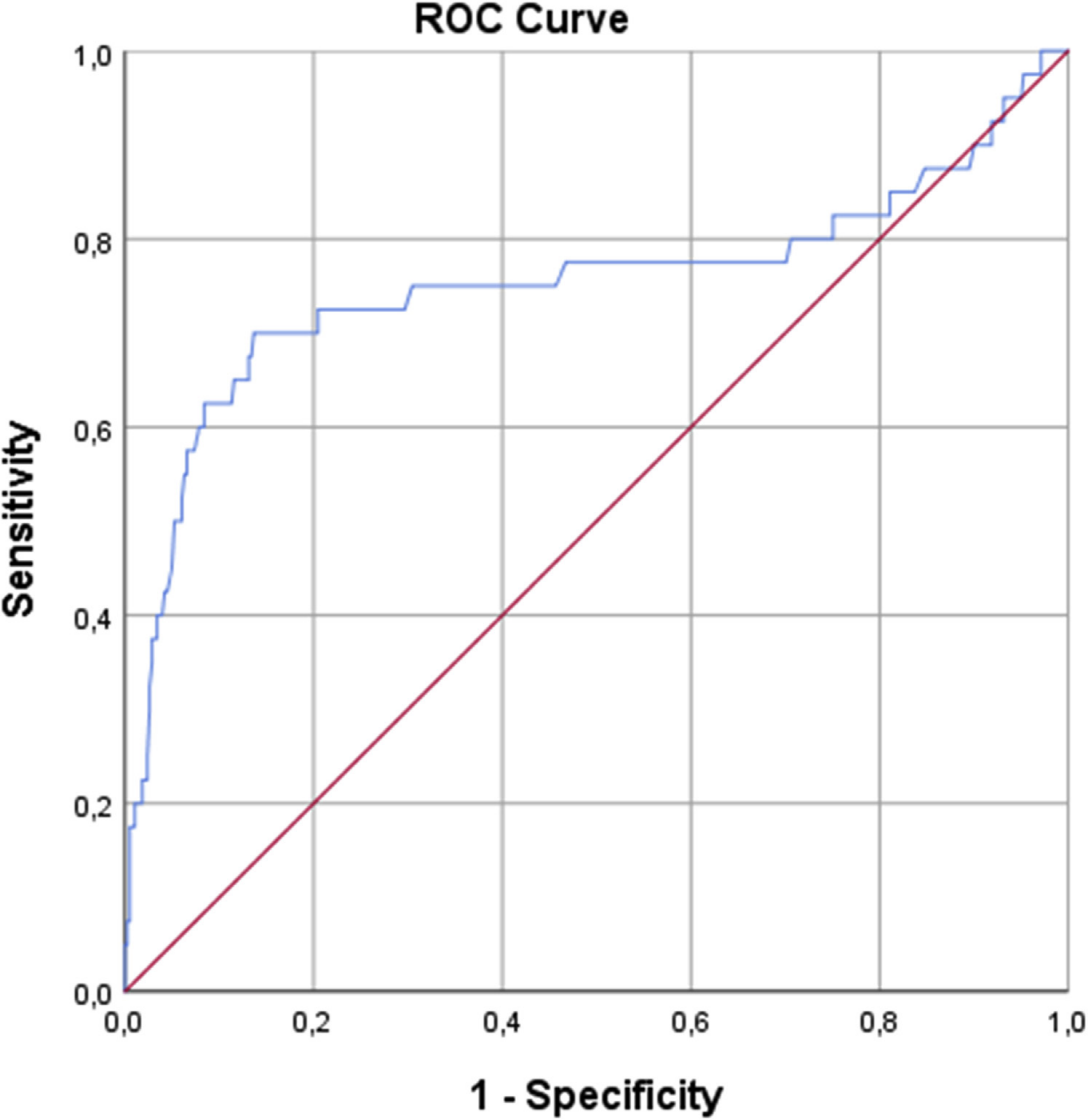
Receiver operating characteristic curve predicting early reconnection/dormant conduction using the 3 parameters.

Multivariable analysis showed that a higher number of unsuccessful freezes (hazard ratio [HR] 1.7 [1.15–2.49], *P* = .008), a longer time to isolation (HR 1.1 [1.01–1.03], *P* = .001), and a higher nadir balloon temperature (HR 1.2 [1.09–1.23], *P* < .001) were independently associated with ERC (Table 4). Male sex was not a significant predictor in the multivariable analyses (*P* = .177). In Figure 1, an ROC curve is constructed with the 3 available parameters with an area under the curve of 0.75. Based on the coefficients—0.02, 0.5, and 0.2 for, respectively, time to isolation, number of unsuccessful freezes, and nadir balloon temperature—a combined cut-off value of ≤-6.7 was calculated to predict ERC with a 86% specificity and 70% sensitivity (negative likelihood ratio 0.35, positive likelihood ratio 5.1, negative predictive value 97%, positive predictive value 34%). By applying the derived formula on different values of the respective parameters, we constructed Table 5 for clinical decision-making to either apply a waiting period with adenosine testing (ERC testing) or refrain from it.

**Table 4:** Biophysical and procedural predictors for early reconnection/dormant conduction

Variables	Univariable		Multivariable	
	Odds ratio (95% confidence interval)	*P* value	Odds ratio (95% confidence interval)	*P* value
**Procedural**				
Time to isolation	1.0 [1.01–1.03]	<.001	1.1 [1.01–1.03]	.001
Number of unsuccessful freezes	1.5 [1.18–2.01]	<.001	1.7 [1.15–2.49]	.008
**Biophysical**				
Temperature at 30 seconds	1.1 [1.02–1.13]	.006	0.9 [0.82–1.0]	.057
Temperature at 60 seconds	1.1 [1.05–1.17]	<.001		
Nadir temperature	1.1 [1.06–1.15]	<.001	1.2 [1.09–1.23]	<.001
Temperature at time-to-isolation	1.0 [0.95–1.04]	.885		
Freeze (area under the curve)	1.0 [1.0–1.0]	.008		
Freeze magnitude	0.9 [0.83–0.93]	<.001		
Warming time to 0°C	0.9 [0.78–0.93]	<.001		
Warming time to 15°C	1.0 [0.94–0.98]	<.001		
Warming time to 20°C	1.0 [0.95–0.98]	<.001

**Table 5:** Cut-off-values for time to isolation to predict early reconnection/dormant conduction

Unsuccessful freezes (n)	0	1	2	3
Balloon temperature (°C)	Time to isolation (s)	Time to isolation (s)	Time to isolation (s)	Time to isolation (s)
-30	ERC test	ERC test	ERC test	ERC test
-31	ERC test	ERC test	ERC test	ERC test
-32	ERC test	ERC test	ERC test	ERC test
-33	ERC test	ERC test	ERC test	ERC test
-34	≥5: ERC test	ERC test	ERC test	ERC test
-35	≥15: ERC test	ERC test	ERC test	ERC test
-36	≥25: ERC test	ERC test	ERC test	ERC test
-37	≥35: ERC test	≥10: ERC test	ERC test	ERC test
-38	≥45: ERC test	≥20: ERC test	ERC test	ERC test
-39	≥55: ERC test	≥30: ERC test	≥5: ERC test	ERC test
-40	≥65: ERC test	≥40: ERC test	≥15: ERC test	ERC test
-41	≥75: ERC test	≥50: ERC test	≥25: ERC test	ERC test
-42	≥85: ERC test	≥60: ERC test	≥35: ERC test	≥10: ERC test
-43	≥95: ERC test	≥70: ERC test	≥45: ERC test	≥20: ERC test
-44	NO ERC test	≥80:ERC test	≥55: ERC test	≥30: ERC test
-45	NO ERC test	≥90: ERC test	≥65: ERC test	≥40: ERC test
-46	NO ERC test	NO ERC test	≥75: ERC test	≥50: ERC test
-47	NO ERC test	NO ERC test	≥85: ERC test	≥60: ERC test
-48	NO ERC test	NO ERC test	≥95: ERC test	≥70: ERC test
-49	NO ERC test	NO ERC test	NO ERC test	≥80:ERC test
-50	NO ERC test	NO ERC test	NO ERC test	≥90: ERC test

Based on the coefficients a formula is created for the 3 significant parameters (0.02 × time to isolation + 0.5 × number of unsuccessful freezes and 0.2 × nadir balloon temperature). With an 86% specificity and 70% sensitivity a combined cut-off-value of -6.7 is predictive for early reconnection. In clinical practice this means that in case of a longer time to isolation than the given numbers, peri-procedural adenosine testing (ERC test) or additional freezing is advised.

ERC = early reconnection/dormant conduction.

### Acute complications and follow-up

In 2 patients groin-related complications (1.3%) and in 4 patients phrenic nerve palsy persisting after 1 year (2.6%) occurred. The 1-year off antiarrhythmic drug AF-free survival in the total group was 69% and was not significantly different between the 2 groups (68% vs 71%, *P* = .983). Eleven patients were lost to follow-up. Reablation was performed in 22 patients, 56% of the patients with AF recurrence (n = 39), and was not significantly different between the ERC and non-ERC groups (20% vs 15%, *P* = .323). In 17 (77%) patients undergoing reablation, PV reconnection was observed. When studying the individual veins in these patients, we observed late reconnection in 3 of 9 (33%) PVs with ERC and in 15 of 77 (20%) veins without ERC. This was not statistically significant (*P* = .388).

## Discussion

### Main findings

The purpose of this study was to identify procedural and biophysical parameters associated with the absence of early reconnection and dormant PV conduction (ERC) during AF ablation with the cryoballoon. We found that unsuccessful freeze attempts, a longer time to isolation, and a higher (warmer) nadir balloon temperature were associated with ERC. Based on these parameters, we constructed an easy-to-use table, which may help to decide for or refrain from a 30-minute waiting period followed by adenosine testing.

### Prognostic significance of dormant conduction

Adenosine testing to reveal and subsequently treat dormant conduction during AF ablation is associated with lower recurrence rates.^5^^,^^9^ An international multicenter randomized study showed an improvement of arrhythmia-free survival using this strategy compared to no adenosine testing, with an absolute risk reduction of 27% and an HR of 0.44 (*P* < .0001).^5^

In addition, applying a waiting period before adenosine testing also appears to be an important tool to detect impending PV reconnections.^10^ We previously could demonstrate that the incidence of dormant conduction was higher after a waiting period of 30 minutes than immediately after PVI. In this study, additional applications for the treatment of dormant conduction resulted in an improved 1-year AF-free survival.^4^ In the current study a comparable outcome between groups with and without ERC was observed after 1 year, regardless of the number of unsuccessful attempts, lower balloon temperatures, and longer time to isolation in the PV. Again this suggests the effectiveness of treating ERC after a waiting period followed by adenosine testing.

The prevalence of dormant conduction can be influenced by the duration of the cryo-application.^2^ In a prior study, we could show that increasing the duration of the cryo-application from 90 to 150 seconds after acute PV isolation resulted in a decreased incidence of dormant conduction from 22% to 4% of the veins.^2^ In the current study, we could demonstrate that dormant conduction after 30 minutes was more prevalent in veins in which more than 1 cryo-application had to be performed to achieve isolation. This may be explained by anatomical properties of the PV ostium causing insufficient PV occlusion and incomplete balloon–tissue contact. Another explanation might be related to the occurrence of edema after the first application, making a second application less effective.^11^^,^^12^ However, although tissue edema (diffuse wall thickening of the antra and ostium of the PVs) is thought to occur immediately after ablation, the time frame of this development is not yet clarified.^13^ Nonetheless, it seems reasonable to perform a single effective freeze instead of an ineffective freeze followed by a second application to treat dormant conduction.^6^

### Biophysical and procedural parameters

There is limited data available about biophysical und procedural parameters and (adenosine-induced) reconnection of the PVs. In a study by Ciconte and colleagues,^14^ which included a relatively small number of patients (n = 50), spontaneous or adenosine-induced reconnection was demonstrated only in 8 PVs (4%). A lower nadir balloon temperature and a longer rewarming time were associated with the absence of acute PV isolation. In another study, a time to isolation ≤65 seconds and a longer-time-cycle integration (which is the integration of time to isolation and the number of freeze cycles) were associated with the absence of acute PV reconnection after a single freeze of 180 seconds and a waiting period of 30 minutes without adenosine testing.^15^ Furthermore, it is shown in patients undergoing a repeat ablation after 14 ± 3 months a shorter time to isolation (≤60 seconds) and a thawing time at 0°C of ≥10 seconds were associated with durable PV isolation.^7^ In a study by Deubner and colleagues,^16^ only the freezing temperature slope (which was strongly correlated with nadir temperature) predicted acute isolation. Reconnection was tested without adenosine after a waiting period of 30 minutes. The authors suggested that this information might be useful to decide for a pull-down maneuver (pulling on the balloon catheter after reaching a temperature plateau to improve contact with the inferior part of the ostium) or aborting a cryoablation. In summary, parameters indicating ineffective contact (time to isolation, a nadir balloon temperature, and the rewarming time) were associated with (adenosine-) induced reconnection. Unlike nadir temperature, temperature at time to isolation was not associated with ERC. We hypothesize that temperature at isolation is influenced mostly by vein diameter, as a larger vein allows the balloon to enter the vein more deeply, which may lead to less cooling by atrial blood and therefore to a lower balloon temperature. In contrast, we hypothesize that nadir temperature is influenced by occlusion grade. With a full occlusion, the temperature will continue to drop during the remainder of the freeze, leading to a lower nadir temperature, whereas in the situation with a small leakage blood from the vein will continuously warm the balloon, leading to a higher nadir temperature. Further studies to confirm this hypothesis are warranted. In the studies analyzing biophysical parameters after a waiting time of 30 minutes, only in the study of Ciconte and colleagues^14^ was adenosine administered. The systematic performance of an additional freeze in case of a nadir balloon temperature of >-35°C may explain the lower rate of observed adenosine-induced reconnections compared to our study. Our results extend the results of these studies, as we found that the number of unsuccessful freeze attempts, the nadir balloon temperature, and time to isolation were the most important predictors of ERC with incorporation of a waiting period of 30 minutes and adenosine testing. A possible explanation why ERC did not predict late recurrence is that ERC was treated with additional ablation. We hypothesize that ERC might be a significant predictor for late reconnection if the 30-minute waiting period with adenosine testing would be omitted.

### Clinical implications

This study identified 3 parameters that can predict the absence of ERC and may be helpful to avoid a waiting period of 30 minutes and adenosine testing in selected patients. Implementing these parameters can shorten the total procedure time. Based on the cut-off values of the 3 parameters, we constructed a table for clinical decision-making that can be easily applied during the procedure to decide for 30 minutes of waiting with adenosine testing. In prior studies, single-parameter cut-off values have been defined for time to isolation and nadir balloon temperature.^17^ As 3 different parameters predicted ERC in our study, we suggested a multivariable prediction model, which was stronger than a single-parameter prediction model in our data. ERC is a non-negligible phenomenon and prediction may be helpful to predict recurrences. Several studies have shown the benefit of treating dormant conduction to improve efficacy.^5^^,^^9^ We therefore feel that it is important to include adenosine testing after cryoballoon ablation. The novelty of our study lies in the prediction model for the absence of ERC after a 30-minute waiting period after a single-freeze protocol, which is the current practice in cryoballoon ablation. Applying our model, operators may decide to refrain from the 30-minute waiting period and adenosine testing in certain patients. Alternatively, operators may decide to immediately perform an additional (bonus) freeze after a suboptimal freeze. The efficacy of the latter strategy should, however, be tested in a future study. In the new version of the cryoconsole, biophysical data will be directly available, which facilitates peri-procedural decision-making. Larger studies, preferably with adenosine testing to reveal dormant reconnection sites, should be performed to develop a model for a cryoballoon ablation score, similar to the ablation index or Lesion Index.^18^^,^^19^ It has been demonstrated that the prospective use of an ablation score system, for example the “ablation index,” can improve clinical outcome.^20^ Similarly, a cryoballoon ablation score may further optimize cryoablation and related outcome. Likewise is AF recurrence in redo procedures, consistent with our study, associated with PV reconnection.^21^

### Limitations

This is a single-center study analyzing procedural and biophysical parameters predicting ERC. The results of this study may be considered as hypothesis-generating and need to be validated in larger cohorts. Without further validation, the reliability of the model and whether it could be used with confidence in clinical practice to eliminate adenosine and a waiting period is unknown. Although the number of acute PV reconnections is low in different reports (varying between 4.5% and 11.1%),^22^^,^^23^ consistent with our results, the number of reconnections including adenosine testing in the current study was 9% of the veins, with 29% of the patients having at least a single reconnected vein. Furthermore, identification and treatment of dormant conduction decreased the rate of AF recurrence in several studies.^5^^,^^9^ We had to use a statistical script to derive the biophysical parameters from text files from the cryoconsole. However, when the new cryoconsole is available, data collection will be easier. This study only investigated the predictors of early reconnection; for a final model it may be important to also analyze data of late reconnection, since this can be dissimilar. The model we constructed shows a modest sensitivity and specificity for the prediction of ERC. In addition, it has not been tested for long-term outcomes. However, with a negative likelihood ratio of 97%, the model is strong enough to exclude patients not requiring adenosine testing. We did not include indirect measurements for balloon occlusion, such as fluoroscopic contrast stasis or intracardiac echo Doppler measurements, into our prediction model. Twenty-four-hour Holter monitoring was used to detect recurrences; longer rhythm monitoring could have detected more AF episodes.

## Conclusion

We identified 3 parameters predicting ERC in cryoballoon ablation: higher nadir balloon temperature, higher number of unsuccessful freezes, and longer time to isolation. From these parameters we constructed a multivariable prediction model. This prediction model may help to avoid a 30-minute waiting period and adenosine testing in selected patients, with a significant reduction of the total procedural duration.

## Funding Sources

This research did not receive any specific grant from funding agencies in the public, commercial, or not-for-profit sectors.

## Disclosures

The Heart Lung Center of the Leiden University Medical Center received unrestricted research grants from Medtronic, Biotronik, Boston Scientific, Lantheus Medical Imaging, St. Jude Medical, Edwards Life Sciences, and GE Healthcare and unrestricted educational grants from Medtronic, Edwards Life Sciences, and St. Jude Medical.

## Authorship

All authors attest they meet the current ICMJE criteria for authorship.

## Patient Consent

Written informed consent was waived owing to the use of retrospective and de-identified data.

## Ethics Statement

This study was approved by the institutional ethical review board. The research in this study was conducted in accordance with the guidelines outlined in the Declaration of Helsinki.
